# Molecular Approaches for High Throughput Detection and Quantification of Genetically Modified Crops: A Review

**DOI:** 10.3389/fpls.2017.01670

**Published:** 2017-10-16

**Authors:** Ibrahim B. Salisu, Ahmad A. Shahid, Amina Yaqoob, Qurban Ali, Kamran S. Bajwa, Abdul Q. Rao, Tayyab Husnain

**Affiliations:** ^1^Department of Animal Science, Faculty of Agriculture, Federal University Dutse, Jigawa, Nigeria; ^2^Centre of Excellence in Molecular Biology, University of the Punjab, Lahore, Pakistan; ^3^Institute of Molecular Biology and Biotechnology, University of Lahore, Lahore, Pakistan

**Keywords:** GMOs, detection, quantification, digital-PCR, micro array, next generation sequencing

## Abstract

As long as the genetically modified crops are gaining attention globally, their proper approval and commercialization need accurate and reliable diagnostic methods for the transgenic content. These diagnostic techniques are mainly divided into two major groups, i.e., identification of transgenic (1) DNA and (2) proteins from GMOs and their products. Conventional methods such as PCR (polymerase chain reaction) and enzyme-linked immunosorbent assay (ELISA) were routinely employed for DNA and protein based quantification respectively. Although, these Techniques (PCR and ELISA) are considered as significantly convenient and productive, but there is need for more advance technologies that allow for high throughput detection and the quantification of GM event as the production of more complex GMO is increasing day by day. Therefore, recent approaches like microarray, capillary gel electrophoresis, digital PCR and next generation sequencing are more promising due to their accuracy and precise detection of transgenic contents. The present article is a brief comparative study of all such detection techniques on the basis of their advent, feasibility, accuracy, and cost effectiveness. However, these emerging technologies have a lot to do with detection of a specific event, contamination of different events and determination of fusion as well as stacked gene protein are the critical issues to be addressed in future.

## Introduction

With the advent of genetic engineering and molecular biology techniques, it has become possible to alter the genome of an organisms through the process termed as transformation. Typically, these alterations involve the insertion of a specific transgenic cassette into the organism’s genome. Usually, the transgenic cassettes consist of elements from species other than the host and contained a desired gene, called a trait which is expressed highly by an upstream strong promoter and became stabilized through a downstream terminator. The genetic expression takes place in two main principal stages: the first step involves the specified gene transcription to messenger RNA. During the second step, the messenger RNA is translated into a protein (Fraiture et al., 2017).

Different products of recombinant DNA technology such as genetically modified (GM) plants (GM corn, GM cotton, and GM soybeans, etc.) and other valuable products like human insulin and growth hormone were already commercially available. Countries such as the United States, Brazil, and Argentina remain the main producer, distributors and sellers of GMO (James, 2014). A lot of issues regarding GMOs testing and confirmation have been raised seriously. Therefore, a reliable approach is required to assess the GM product quantitatively before it’s commercialization and to regulate unofficial utilization of the transgenic events (Amiri et al., 2013).

Analytical techniques for GMO detection fall into two main categories: the indirect method (protein-based detection method) or direct method (DNA-based detection methods) (Randhawa et al., 2016). Conventional PCR has been used ideally for detection of both raw and processed GM products, but the advent of recent advancements in biotechnology such as microarray, capillary gel electrophoresis (CGE), loop-mediated isothermal amplification, digital PCR, and next generation sequencing has updated the detection method to a remarkable point (Milavec et al., 2014). Very few reports are available that have paid attention to address the recent approaches in detection and quantification of GMO. The present article is, therefore aimed at providing an overview of the most commonly used GM diagnostics techniques along with recent advances in this field.

## General Considerations for Detection of Transgenic DNA and Novel Protein from GMO

Various universal considerations such as sample preparation, food matrix effects on either protein or DNA extraction are required for application of GMO identification techniques (i.e., protein or DNA based). Parameters for example Reference materials, validation of technique, standards harmonization as well as the accessibility to the collection of organized information also remained valuable for proper implementation of these techniques. Sampling is very crucial as the determination primarily depends upon the GM material from which the sample is obtained. Factors such as sample heterogeneity and sample size need to be taken into consideration during sampling (Bertheau et al., 2002; Trapmann et al., 2002). The techniques applied to extract DNA or proteins from the sample have also been considered to play a key role toward reducing the chances of error in results interpretation (Alexander et al., 2007; Ishfaq and Saleem, 2016).

## Quantitative GM Detection

Most recent detection approaches depend either on the PCR (polymerase chain reaction) technology to amplify transgene sequence(s) or on immunological techniques mainly ELISA (the enzyme-linked immunosorbent assay) to bind to a transgene gene product(s) (Tan et al., 2013; Randhawa et al., 2016). Though, specific DNA sequences can also be identified through hybridization, it is PCR in its different formats (qualitative PCR, end-point quantitative PCR, and quantitative real-time PCR) which has been widely recognized by the regulatory authorities (Marmiroli et al., 2008). All PCR technologies require that, a minimum amount of known target DNA sequences to be present in the DNA template. The extraction of the DNA and its purification from the sample matrix is the most crucial step (Cankar et al., 2006). PCR technology remained the most popular and reliable molecular technique for primary screening of GMO to detect the presence of specific DNA sequence from samples even with very less or poor DNA quality. This technology has been widely used due to its flexibility, sensitivity, specificity as well as the applicability to wide range of materials.

The PCR-based GM testing technologies have been partitioned into four groups based on the variations between the various integrated exogenous elements namely (i) screening methods (ii) gene (iii) construct and (iv) event-specific methods (Li et al., 2012). Screening of GMO involves detection of regulatory elements primarily associated with GMO (i.e., promoter and terminator sequences) (Forte et al., 2005). The transgene-specific method identifies a particular gene, for instance, EPSPS (herbicide tolerance) or *Cry1Ab, Cry9c* (insect resistance) while construct-specific technique aims at sequence flanked by two DNA elements obtained in a specific construct of a transgene, e.g., promoter and gene. Different studies have also shown that various target genes (*ctp2- cry2Ab2, ctp2-cp4epsps, p35S-cry1Ac, p35S-uidA*), could be detected by construct-specific techniques (Grohmann et al., 2009; Chhabra et al., 2014). Event-specific PCR detection technology is commonly employed for GMO testing due to its ability to specifically detect each transgenic event simply by targeting their unique junction between the host genome and the transgenic cassette (Zhang et al., 2015). Currently, different event-specific Q-PCR (quantitative) technology has been designed for transgene detection from GM Corn, Cotton, Canola, Rapeseed, and rest of the crops (Lee et al., 2009; Wu et al., 2009; Jiang et al., 2010).

Quantitative real-time PCR (q-rtPCR) has remained the most reliable method for GMO quantification. This technology has presented several advantages over the conventional PCR analysis as the amplification of DNA occurred in real time. Moreover, the starting DNA concentration in q-rtPCR is obtained with accuracy and greater sensitivity. The real-time results can either be qualitative or quantitative. In contrast, traditional PCR is semi-quantitative at its best. Furthermore, the products of q-rtPCR are analyzed in a closed-tube system, bypassing the post-amplification modifications and therefore, reducing the risk of contamination (Navarro et al., 2015). Despite the advantages offered by q-rtPCR technology over conventional PCR, its success largely relies on various factors, e.g., its throughput strategy is often restricted to one marker per reaction. Due to continuous growth in GMO production, new/additional detection markers (for specific detection of new transgene) are required to be designed continuously and used to completely cover their identification. This will possibly turn the experimental process more difficult and tedious as well (Broeders et al., 2012). To overcome these issues, novel alternative approaches have been designed which allows for better as well as quick detection of GMO both in field and lab condition (Fraiture et al., 2015).

## Novel Approaches for GMO Detection and Quantification

Due to the continuous increase in production and complicacy of GMO carrying both single and multiple genes insert, transgenic detection, especially for each single event, is becoming laborious and expensive (Novak et al., 2009; Holst-Jensen et al., 2012; Žel et al., 2012). Transgenic events having only a single trait can be detected by employing a simple conventional PCR technique, whereas detection and quantification of GM events with multiple or stacked traits require the application of the combination of high-throughput technologies (Randhawa et al., 2016). With the recent advances in molecular biology and keeping in mind the limitations of conventional methods, new techniques have been developed for DNA-based detection of GMO which aimed at improving the standard of traditional qPCR as well as time-consuming gel electrophoresis (Brod et al., 2014). These novel approaches are so reliable for the quantification and detection of specific transgenic events thus provide the solution to some of the problems that are associated with currently used techniques (Milavec et al., 2014). Previously, different alternative techniques which used the various extension and detection strategies were designed for GMO identification (Holst-Jensen, 2009; Žel et al., 2012). Currently, more reliable and promising methods were designed (Shao et al., 2014). Not all the newly designed technique can be applied for multi-targeting or multiplex quantification (Milavec et al., 2014). Therefore, some of the most productive approaches are discussed below:

## Capillary Gel electrophoresis (CGE)

**FIGURE 1:**
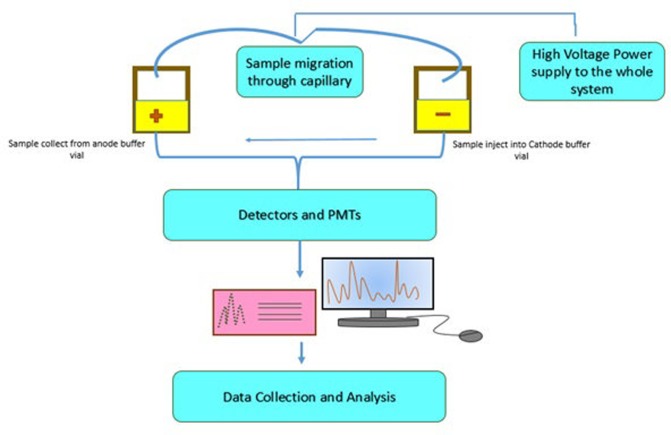
General work flow for capillary gel electrophoresis (CGE). Source: Kerékgyártó and Guttman (2015).

Heide et al. (2008b) proposed a technique that can be used to identify various transgenic events in one reaction. A nine-plex (9-plex) system coupled with identification via PCR-CGE was developed by Heid and his co-workers (**Figure 1**). The basic principle behind this technique is to carry out multiple PCR reaction using forward primers which are fluorescently labeled and discrimination of amplimer of similar magnitude by executing CGE. The technique is mainly designed for the transgenic event detection from GM corn. As compared to the electrophoresis gel, CGE system has higher resolution power to clearly detect PCR products from a multiplex assay (Vega and Marina, 2014). Moreover, a single5-plex (pentaplex) PCR and double 6-plex (hexaplex) PCR have also been designed specially to identify different numbers of events from GM corn and cotton (Nadal et al., 2009; Holck and Pedersen, 2011). Currently, researchers have also reported event specific identification of cotton by using 4-plex (tetraplex) detection technology (Basak et al., 2014). Additionally, three 8-plex (Octaplex) PCR system is also developed. This system employs universal tailed primers which pre-amplify the desire sequences within few cycles (Guo et al., 2011). In order to increment the quantity and quality of PCR, universal primers must be supplied to these amplimers. Through this strategy, a number of transgenic events have been identified by means of CGE technology. Another form of this method, which does not indicate the use of fluorescent labels upon primers has been proposed recently (Burrell et al., 2011). The research has suggested a 4-plex (tetraplex) PCR system having two gene -specific methods and a double marker gene for screening which allow the identification of transgenic events from GM corn (Bt11 gene) GM soybean (*GTS40-3-2* gene) employing commercial electrophoresis devices (**Table 1**).

**Table 1:** Multiplex PCR-CGE techniques for detection of GMO.

Multiplexing	Techniques	Target genes	Reference
4-plex	Taxon specific	*LEC, Zein*	Burrell et al., 2011
	Element specific	*p35S, tNOS*	
5-plex	Taxon specific	*ADH*	Holck and Pedersen, 2011
	Event specific	*GA21, MON810, NK603, Bt11*	Nadal et al., 2009
6-plex	Taxon specific	*acp1*	Guo et al., 2011
	Event specific	*Bollgard, Bollgard II, RR, 3006-210-23, 281-24-231*	
9-plex	Element specific	*bar, chy, pAct, CP4-EPSPS, Cry1Ab*	Guo et al., 2011
	Event specific	*GT73, OXY235*	
9-plex	Taxon specific	*HMG*	Heide et al., 2008b
	Event specific	*T25, GA21, TC1507, MON863, MON810, NK603, Bt176, Bt11*	

*Zein, Zein gene obtained from corn; *LEC*, soybean lectin gene; *p35S*, 35S cauliflower mosaic virus promoter; *tNOS*, nopaline synthase gene terminator; *ADH*, alcohol dehydrogenase I gene obtained from corn; *Chy*, chymopapain gene of papaya; *acp1*, acyl carrier protein 1 gene of cotton; *bar*, phosphinothricin-*N*-acetyltransferases gene of *Streptomyces hygroscopicus; HMG*, major high-mobility group protein gene of corn; *pAct*, rice actin gene promoter section; *CP4-EPSPS*, 5-enolpyruvylshikimate-3-phosphate synthase gene of *Agrobacterium tumefaciens* strain; *Cry*, gene containing *Bacillus thuringiensis* δ-endotoxin*.

However, CPG has some disadvantages as it requires extensive labor for designing of primer as well as the optimization when performing the analyses for detection of a new event. Its implementation also requires specialized apparatus which may not always be available. Since the technique is not commonly employed in the quantification of transgenic events, thus there is need of its absolute authentication and confirmation (Milavec et al., 2014; Fraiture et al., 2015).

## Loop mediated isothermal amplification (LAMP)

Loop mediated isothermal amplification (LAMP) is also an emerging technology which was developed toward quick as well as easy detection of the transgenic event in a given sample (Chen et al., 2012; Li et al., 2013). The main idea behind this technology is the amplification and identification of the desired nucleic acid sequences at a steady temperature and at some specific stage of the experiment (Randhawa et al., 2016). The technique was developed for identification of different types of Bt-transgenic event from rice (**Table 2**) (Milavec et al., 2014). This novel approach of GMO detection involves the utilization of four distinct primers which identify at least six different segments of the desired DNA. The reaction is initiated by one of the DNA primer pair having both similar and opposite sequences of the desired nucleic acid template. The reaction progressed isothermally and then another primer aid in the formation of loop structure which increases both specificity and speed of the amplification. The reaction process is completed in a single tube by employing simple equipment at a constant temperature (Tomita et al., 2008; Fraiture et al., 2015). A ladder -like structure usually indicate the LAMP product on the agarose gel, and sometimes the amplification of the product is visualized in real-time through employing turbidimetry or fluorescent detection by using real-time LAMP (Mori et al., 2001; Randhawa et al., 2013; Huang et al., 2014). The amplified products can also be observed alternatively at the end of LAMP reaction through the utilization of fluorescent dyes, for instance, SYBER Green or using nucleic acid staining (Guan et al., 2010; Chen et al., 2012). Varieties of LAMP markers were thus designed for quantitative detection of transgenic GM events (**Table 2**) (Cheng et al., 2014; Zahradnik et al., 2014).

**Table 2:** Simplex LAMP strategies for the detection of GMO.

Techniques	Target gene	Reference
Taxon specific	*ADH*	Kiddle et al., 2012
	*LEC*	Di et al., 2014
	*PLD, IVR*	Chen et al., 2011
Element specific	*Cry1Ab, CP4-EPSPS*	Wang et al., 2015
	*Cry1Ac*	Li et al., 2013
	*Cry2A, Cry3A*	Zhang et al., 2012
Construct specific	*p35S/EPSPS*	Lee et al., 2009
Event specific	*Bt11, Bt176*	Chen et al., 2011
	*MON863, TC1507*	Zhang et al., 2012

*ADH, alcohol dehydrogenase I gene from corn; *LEC*, soybean lectin gene; *PLD*, phospholipase D gene of rice; *IVR*, invertase gene from corn; *Cry*, gene carrying *Bacillus thuringiensis* δ-endotoxin; *CP4-EPSPS*, 5-enolpyruvylshikimate-3-phosphate synthase gene of *Agrobacterium tumefaciens* strain; *p35S*, 35 S cauliflower mosaic virus promoter*.

Recently, LAMP technologies are being utilized mainly for qualitative determination in various field of diagnostics as a result of their simplicity, time-efficiency and ability to withstand different PCR inhibitors, for instance, acidic polysaccharides (Zhang et al., 2012). Simple devices for instance: water bath and heating block are required for its implementation (Cheng et al., 2014). However, this technique has some limitations, i.e., limitations in designing four primers per sequence. Furthermore, detection of different GM events employing multiplex approach is also a problem (Angers-Loustau et al., 2014). Another serious issue with LAMP assay is the difficulty in result determination when the amount of LAMP products is very low (Di et al., 2014). LAMP assay is mainly quantitative. However, scientist have reported an attempt for qualitative detection of GM events Using LAMP (Soleimani et al., 2013; Huang et al., 2014).

Quantitative LAMP assay aimed at detection of the transgenic event more precisely and holds the potential to replace the existing techniques but need to develop further.

## Digital PCR (dPCR)

In order to overcome some of the problems experienced during qPCR approach particularly in the presence inhibitors or least transgene copy numbers, dPCR is proved to be an excellent technology (**Table 3**). It is one of the most reliable techniques among the currently used technology for GMO quantification. The basic idea behind this novel technology is the quantification of the desired events found in GMO sample by applying limited dilutions and Poisson statistics following PCR (Milavec et al., 2014). The process is accomplished through dividing the mixture of PCR into a sizeable amount of distinct reactions which include null, single or least target DNA copies. After completion of PCR, the positive (i.e., observed replicated desired segments) and negative (i.e., observed unreplicated segments) samples are analyzed and then the total copy number of the desired gene in an original sample is determined by the application of binomial Poisson statistics (Pinheiro et al., 2011; Fraiture et al., 2015).

**Table 3:** Representative examples indicating the dPCR approaches detecting GMO.

Multiplexing	Techniques	Methods	Target genes	Reference
Simplex	cdPCR	Taxon specific	*HMG, LEC, GLU, and CRU*	Brod et al., 2013
		Element specific	*Cry1Ab, Cry1F, Cry1A.105, and Cry2Bb*	Brod et al., 2014
	ddPCR	Event specific	*Bt176, Bt11, GA21, and GT73*	Morisset et al., 2013;
		Taxon specific	*HMG*	Li et al., 2015
		Event specific	*MON810*	
Duplex	cdPCR	Taxon specific	*HMG*	Corbisier et al., 2010
		Event specific	*MON810*	Burns et al., 2010
	ddPCR	Taxon specific	*HMG*	
		Even specific	*MON810*	Morisset et al., 2013

*HMG, major high mobility group protein gene corn; *LEC*, soybean lectin gene; *GLU*, sugar beet glutamine synthetase gene; *CRU*, cruciferin gene of colza; *Cry*, gene carrying *Bacillus thuringiensis* δ-endotoxin*.

Two types of dPCR systems are presently available (Hindson et al., 2011; Pinheiro et al., 2011). Chamber (c) dPCR is one of the kinds of PCR system in which microfluid chambers having many divisions (up to a few 1000) of individual reactions is used. By using this technology transgenic event (MON810) from GM corn was successfully detected and the limit of its detection is being studied as well (Burns et al., 2010). Droplet (d) dPCR is the other type of dPCR system in which utilizes the water–oil emulsion having several divisions (sometimes up to millions) of single droplets which have been analyzed by employing flow cytometry system of analysis. This system was applied for detection of corn event as well (MON810) (**Table 3**) (Morisset et al., 2013; Li et al., 2015). Although, these two detection approaches (cdPCR and ddPCR) have a similar estimation of absolute copy number, yet the measurement uncertainty is greater for cdPCR (Milavec et al., 2014). Besides, ddPCR depend on fluorescence end point identification or extended target, while cdPCR tracks the extension at present situation just as in the case of qPCR. Though, dPCR is being employed already for variety of applications, and mainly in experiments that involve the detection of absolute copy number (Hindson et al., 2011; Sanders et al., 2011), it has been shown as a special technology which is applicable for identification of rare and less copy number targets (Bhat et al., 2009), to evaluate differences in copy number for instance fractions of 1.25, or even less than 1.2, could be differentiated (Weaver et al., 2010; Whale et al., 2012).

In term of quantification of the transgenic event, dPCR has many advantages when compared with qPRC. dPCR allow for detection of target copy numbers contained in a given reaction in an absolute manner, therefore, the preference of extension efficiency among samples and reference material during qPCR is totally avoided (Bhat et al., 2009; Corbisier et al., 2010; Morisset et al., 2013). The information obtained from dPCR are so accurate and provide promising results, which are very valuable for the metrological application (Bhat et al., 2009; Corbisier et al., 2010; Morisset et al., 2013). Furthermore, quantitation using dPCR also allow for the correct estimations of targets even at least copy numbers (Whale et al., 2013). Another striking quality of dPCR is its flexibility in assays transfer from qPCR to dPCR mode, this enables the laboratory implementation of the dPCR much simpler when compared to other techniques (Milavec et al., 2014).

Digital PCR has also shown to be cost-effective especially the ddPCR which has proven to be more appropriate for continuous utilization in control laboratories, particularly when large number of samples need to be dealt with (Morisset et al., 2013). When ddPCR is to be compared with cdPCR, in terms of cost of operation, the device used for cdPCR is more expensive than the one used for ddPCR, and the employed arrays in cdPCR are comparatively costlier. For incrementing the performance price of dPCR, multiplexing system could be employed (Milavec et al., 2014). In a lone reaction, dPCR systems allow for minimum multiplexing of two targets and maximum of 10 targets. Extension by multiplexing largely depends upon the application of probes which are variously labeled and having about five and two distinct fluorophores utilized in both cdPCR, and ddPCR respectively. Multiplexing for up to 10 targets in a single reaction is also possible with by employing primer or probe concentrations (Fraiture et al., 2015). Present developments in ddPCR technology have made it possible to utilize the DNA-binding dye chemistry, which also enables multiplexing (McDermott et al., 2013). In a nutshell, ddPCR is currently considered to be the most reliable technique for perfect quantitation of transgenic event in a given sample due to its wider coverage linearity in quantitation and its greater effectiveness in cost.

## Microarray technology

Microarrays also termed as DNA chips or biochips. It is an advanced technology for high-throughput detection of GMO. This technology, parallel detection of a large number of genetic elements from complex DNA samples in a single assay can be achieve with high septicity. As a highly advance technique, it can evolve together with the growing number of newly developed GMO in the food and feed markets. Miniaturization, high sensitivity and screening throughput are the major advantages of this technology (Turkec et al., 2016). These attributes allow for not only samples analysis for detecting the existence of transgene (individual or selected group) or control genetic elements, but also to increase many probes analysis in a single hybridization study (Fraiture et al., 2015). The principal idea is that numerous designated probes get bound onto a solid surface in a spot-wise in array manner with individual spot having many duplicates of the probe. The Isolated DNA of the desired sample that is being hybridized with an array is then marked fluorescently. At hybridization stage, the marked segment of DNA remains combined with the spotted probes based on the opposite DNA sequences. The greater the length of opposite DNA sequences tougher the bond will be. Following hybridization phase, sequences which are poorly bound to the probes together with the residual free marked sequences are removed and then scanned the array to check the intensity of the individual fluorescence of each spot. The major advantages of DNA chips are miniaturization, and high-throughput screening (Randhawa et al., 2016).

DNA chip technology coupled with multiplex PCR can be used in the identification of different transgenic events from GMOs by employing multiplex PCR approaches (**Table 4**) (Marmiroli et al., 2008). When compared to qPCR, DNA chip technology provides better result with a higher throughput but somewhat less in sensitivity (Pla et al., 2012). Various detection strategies combined with multiplex PCR were being reported (**Table 6**) (Hamels et al., 2009). Nucleic acid array in combination with multiplex PCR has been used successfully for identification of different types of events from GM crops like corn and cotton (Leimanis et al., 2008; Kim et al., 2010; Fraiture et al., 2015). Transgenic events from GM corn were also identified using MQDA-PCR (multiplex quantitative DNA array based) approach. This technology involves the use of gene-specific PCR primer. The primer harbored a common tail which allows the re-use of primer for the subsequent PCR. Following completion PCR process, the signal is then observed after the hybridization of the amplified products with probes which are marked fluorescently on the DNA array (Fraiture et al., 2015). Similarly, scientists have reported the detection of GM events from maize, cotton, and soybean by employing PPLMD (padlock probe ligation microarray detection) system (Prins et al., 2008). Additionally, a study has also shown the possibility of detection of GM event from corn using another detection technology called NAIMA (nucleic acid sequence based amplification implemented microarray) system.

**Table 4:** Multiplex PCR microarray approaches for detection of GMO.

Multiplexing	Techniques	Methods	Target genes	Reference
2-plex	Dual Chip	Element-specific	*p35S and tNOS pNOS/nptII*	Hamels et al., 2009
	GMO	Construct-specific		
3-plex	NAIMA	Element-specific	*p35S MON810*	Dobnik et al., 2010
		Event-specific		
4-plex	Dual Chip GMO	Taxon-specific	*IVR, LEC, and CRU*	Milavec et al., 2014
8-plex	MQDA-PCR	Element-specific	*p35S and tNOS Bt176*,	Fraiture et al., 2015
		Event-specific	*Bt11, and MON810*	
10-plex	PPLMD	Element-specific	*p35S, pFMV, and*	Prins et al., 2008
		Event-specific	*bar MON1445, Bt176*,	
		Taxon-specific	*and GTS40-3-2 HMG*	

*p35S, 35 S cauliflower mosaic virus promoter; *tNOS*, nopaline synthase gene terminator; *pNOS*, nopaline synthase gene promoter; *nptII*, neomycin phosphotransferase II gene; *IVR*, invertase gene of corn; *LEC*, soybean lectin gene; *CRU*, cruciferin gene of colza; *pFMV*, promoter of the figwort mosaic virus gene; *bar*, phosphinothricin*-N* acetyltransferases gene of *Streptomyces hygroscopicus; HMG*, major high mobility group protein gene of corn*.

**FIGURE 2:**
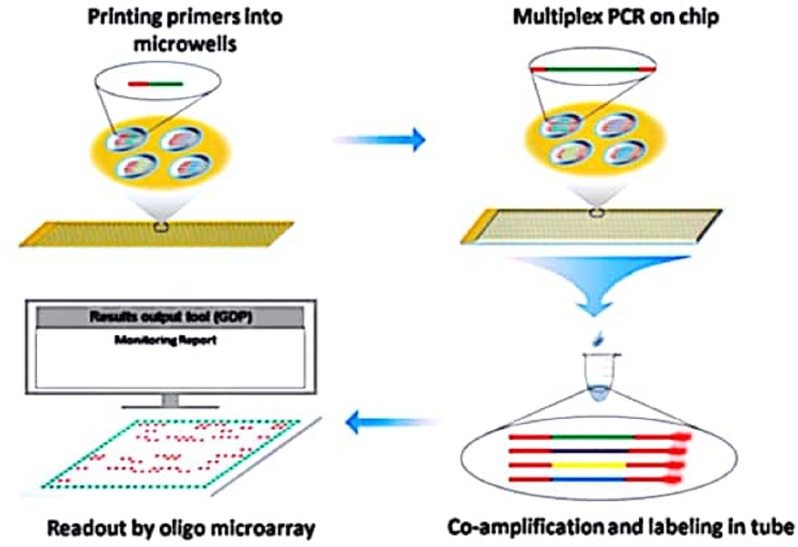
Schematic diagram representing coupling of Microchip PCR and Microarray System for High throughput events detection from GMO. Source: Shao et al. (2014).

This technology employed tailed primers that allow for the multiplex production of DNA template in a primer extension reaction, as well as the subsequent transcription-based extension using common primers (Dobnik et al., 2010). Dual Chip GMO system was also suggested as a substitute to the likely problem in respect of fluorescent label utilization. By using this approach, simultaneous detection of GM maize, soybean and rapeseed events is possible through colorimetric reaction following PCR amplification with biotinylated target specific primers (**Table 4**) (Milavec et al., 2014). Furthermore, Shao and his co-workers (**Figure 2**) also reported a multiplex extension on a microarray having data on an oligo microarray (MACRO) system, aiming ninety-one targets for a wider range detection coverage of GM events (Shao et al., 2014).

## Next generation sequencing (NGS)

Next generation sequencing is a novel technique which is recently proposed with an aim of dealing with the challenges linked with detection of transgenic events of GMOs. It is a promising technology that allows for massively parallel DNA segment sequencing resulting in millions of sequencing read (Willems et al., 2015; Fraiture et al., 2017). NGS is an efficient tool for transgenic events detection even in the absence of sequence information of such events (Randhawa et al., 2016). The technique has been used generally for mutant-site detection (Polko et al., 2012), Analysis of Nucleic Acid expression profile (Fullwood et al., 2009), and copy number variations in humans, plants, animals as well as in micro-organism, with the greater benefit of excellent quality, accuracy and satisfactory information at the complete level of the genome (Campbell et al., 2008; Hormozdiari et al., 2011). Various research trials have been done so far to check the application of NGS in GM content determination. NGS is being efficiently employed for characterization of site addition, flanking regions, accidental addition as well as the determination of transgene copy number (Milavec et al., 2014). Two main approaches (targeted sequencing strategy) or [whole genome sequencing (WGS) strategy], for samples sequencing which has been enriched previously with desire sequence regions have been identified (**Table 7**) (Fraiture et al., 2017).

The targeted sequencing approach is particularly useful for sequencing the desired gene regions from both large and intricate genomes, found mostly in plants. This sequencing strategy offers an advantage to exclusively utilize all the energy, with respect to time and cost on the regions of interest (i.e., it saves time and cost). Another striking advantage is that it requires less previous sequences information in order to sequence the desired gene fragments (Fraiture et al., 2015). From this approach, dual sub-strategies can be employed. One of the sub-strategies is the amplicon sequencing (i.e., sequencing of the DNA library of PCR products). The other second sub-strategy is the target enrichment sequencing (i.e., sequencing of the selected DNA segments from a complete genome library) (Liang et al., 2014; Song et al., 2014). By using this technology, transgenic events identification from GM corn (*vip3A gene in MIR162*) and cotton (*Bt11*) has been successfully achieved. Region identification PCR coupled with NGS aiming the gene region of the vip3Aa20 element in MIR162 is being demonstrated (Liang et al., 2014) (**Table 5**).

**Table 5:** Representative examples indicating NGS strategies targeting transgenic event.

NGS approaches	NGS platforms	Target genes	Target sizes	Reference
Targeted sequencing	454 systems (Roche	*Bt11*	324 bp	Song et al., 2014
	Applied Science)	*CP4-EPSPS*	498 bp	
		*LEC*	118 bp	Song et al., 2014
		*p35S*	195 bp	Song et al., 2014
	PacBio RS (Pacific	*vip3Aa2 from*	150 bp to	Song et al., 2014
	Biosciences)	*MIR162*	2 Kbp	Liang et al., 2014
Whole genome Sequencing		*LLRICE62 rice*	385 Mbp	Wahler et al., 2013
		*Bt rice*	385 Mbp	Willems et al., 2016
	HiSeq (Illumina)	*TT51-1 rice*	385 Mbp	Yang et al., 2013
		*MON87704 soybean*	1115 Mbp	Yang et al., 2013
		*MON17903 soybean*	1115 Mbp	Kovalic et al., 2012

*CP4-EPSPS, 5-enolpyruvylshikimate-3-phosphate synthase gene from *Agrobacterium tumefaciens* strain; *LEC*, soybean lectin gene; *p35S*, 35S cauliflower promoter; *VIP3A*, vegetative insecticidal protein 3A*.

**Table 6:** General properties of high-throughput techniques employed for GMO detection.

Technique	Sensitivity	Specificity	Quantification	Multiplexing	Amplification time (mnt)	Tested sample	Amplification method	Detection method	Reference
rt-PCR	5 (0.1%)	Yes	Yes	1 (2)	100	P, S F, F_0_	PCR	Real time	Pla et al., 2012
2S-PCR-CGE	40	Yes	Yes	9	240	S, F	PCR	Capillary gel electrophoresis	Heide et al., 2008a
ddPCR	<5	Yes	Yes	10	100	S, P_o_	PCR	End-point flow cytometry	Morisset et al., 2013
cdPCR	<5	Yes	Yes	5	100		PCR	Real time/end point flow	Burns et al., 2010
MQDA	10	Yes^a^	Yes	12	100	S, F, F_0_	PCR	Microarray	Rudi et al., 2003
PPLMD	13 (0.1%)	Yes	Yes	10	100	P, S	PCR	Microarray	Pla et al., 2012; Ujhelyi et al., 2012
NAIMA	10 (0.1%)	Yes	Yes	3 (6^b^)	25–45	P, S F, F_0_	NASBA	Microarray	Dobnik et al., 2010; Pla et al., 2012

*rt-PCR, real time PCR; 2S- PCR-CGE, two-step PCR combined with capillary gel electrophoresis; ddPCR, droplet digital PCR; cdPCR, chamber digital PCR; MQDA-PCR, multiplex quantitative DNA array-based PCR; PPLMD, padlock probe ligation and microarray detection; NASBA, nucleic-acid-sequence-based amplification; NAIMA, nucleic-acid implemented microarray analysis. Sensitivity: least detectable number of target copies given in absolute copy number [absolute limit of detection (aLOD); first number] and relative LOD (rLOD; in brackets). Specificity: as suitable for detection of GMO. Quantification: as suitable for GMO detection. Multiplexing: given as the maximum number of concurrent amplifications in single reaction. Amplification time: rough estimates only in minutes. Tested sample: P, plant material; P_*o*,_ plasmid DNA; S, seed or seed flour; F, food sample; F_*0*,_ feed sample. ^a^Only as semi quantitative, ^b^preliminary result (data unpublished).*

**Table 7:** Common useful and limited properties for quantitative techniques used for GMO diagnostics.

Technologies	Advantages	Limitations	Reference
rtPCR	Faster, highly specific, allow multiplexing and permit quantification	One marker per reaction	Broeders et al., 2012; Navarro et al., 2015
CGE	Specificity, sensitivity, multiplexing and quantification a, higher resolution power to clearly detect PCR products from a multiplex assay	Extensive labor for primer design and optimization, specialized apparatus is required	Heide et al., 2008b; Milavec et al., 2014; Vega and Marina, 2014; Fraiture et al., 2015
LAMP	Required simple devices, time-efficiency, ability to withstand different PCR inhibitors	Four primers per sequence	Zhang et al., 2012; Angers-Loustau et al., 2014; Cheng et al., 2014
dPCR	Multiplexing, flexibility, absolute detection of target copy number, accurate estimation of target at low copy number	Relatively expensive	Hindson et al., 2011; Sanders et al., 2011; Milavec et al., 2014
Microarray	Miniaturization, multiplexing, high-throughput screening	Difficulties in prove designing, data processing is laborious	Pla et al., 2012; Randhawa et al., 2016
NGS	No prior sequence information is required, high accuracy, direct sample identification, time-efficiency	Relatively expensive, requires sophisticated devices, data analysis issues	Buermans and Den Dunnen, 2014; Randhawa et al., 2016; Willems et al., 2016

*rtPCR, real time PCR; CGE, capillary gel electrophoresis; LAMP, loop mediated isothermal amplification; dPCR, digital PCR; NGS, next generation sequencing*.

The NGS approach enables in principle characterizing a sample in the absence of sequence information. Using this sequencing technology, the whole DNA library having constructs of genomic DNA with adaptors is sequenced. The generated reads are allowed to treat with bioinformatic tools for the purpose of GM correlation with already available data (Yang et al., 2013). Molecular analysis of transgenic varieties from GM soy and GM rice (Kovalic et al., 2012; Wahler et al., 2013; Yang et al., 2013) have successfully been achieved using this strategy (**Table 5**). The NGS technology has proven to be an alternative in the area of GMO detection as it provides the chance of direct identification for GM presence in a given sample through the characterization of their sequences. In addition, new PCR markers could be designed from the sequences detected for identification of the unknown GM events. However, this technology is relatively expensive and requires sophisticated devices as well as a bioinformatic analyst for manipulation and analysis of obtained data. This, of course, makes its implementation difficult (Buermans and Den Dunnen, 2014; Willems et al., 2016). It is anticipated that this novel technology will become more sensitive and more suitable and that could provide a more promising solution for the recent challenges of GMO analysis in the near future (Milavec et al., 2014).

## Conclusion

With continuous growth in production of GMO as well as diversification of traits worldwide, there is a need for cost-effective GMO testing that will possibly simplify the efficient evaluation of hazards, management as well as monitoring following their release, in order to diminish public fear and resolve authorized disputes. Different molecular technologies are now available for evaluating the absence or presence of GMO in samples, and for their detection as well as quantification. However, time-consuming conventional PCR and ELISA based methodologies are replaced by the recently highly fast and convenient technologies which are now approved globally for GM detection. In the near future, it is anticipated that these recent approaches having the capability of absolute quantification and generating large amounts of information in a single experiment will get their proper position in the world of GMO identification and quantification.

## Author Contributions

The author IS write up the initial draft of manuscript under the supervision of AS. AY edited the manuscript for minor corrections. QA make final version of the manuscript after important changes. KB proof-read the manuscript while AR and TH gave final approval for publication of manuscript.

## Conflict of Interest Statement

The authors declare that the research was conducted in the absence of any commercial or financial relationships that could be construed as a potential conflict of interest.
